# Predictive markers for clinical outcomes in a cohort of diabetic patients hospitalized for COVID-19

**DOI:** 10.1186/s13098-022-00941-7

**Published:** 2022-11-12

**Authors:** Sara Madaschi, Eugenia Resmini, Silvia Bonfadini, Giulia Massari, Paola Gamba, Marco Sandri, Stefano Calza, Elena Cimino, Emanuela Zarra, Silvia Dotti, Cristina Mascadri, Barbara Agosti, Emirena Garrafa, Angela Girelli

**Affiliations:** 1 UOC Medicina Generale ad indirizzo Metabolico e Diabetologico, ASST degli Spedali Civili di Brescia, Brescia, Italy; 2 Department of Molecular and Translational Medicine, University of Brescia, Brescia, Italy; 3 ASST Spedali Civili di Brescia, Department of Laboratory,, Brescia, Italy

**Keywords:** Diabetes mellitus, Inflammation, COVID-19, Hyperglycemia, Clinical outcomes

## Abstract

**Introduction:**

The role of glycemic control, both prior and during hospitalization, on mortality from COVID-19 in diabetic patients is debated. Furthermore, it is not clear whether hyperglycemia has a direct effect or requires inflammatory mechanisms.

**Objective:**

To identify predictors of clinical outcomes (in-hospital mortality, length of hospitalization, respiratory failure, need for intensive care), considering hyperglycemia, inflammation markers and clinical history.

**Methods:**

Retrospective observational study of 291 diabetic patients hospitalized with COVID-19 in the Spedali Civili di Brescia from February 1th 2020 to March 31th 2021, with also outpatient electronic records. Glucose, inflammatory parameters, creatinine were collected within 24 h after admission to the hospital. A causal mediation analysis allowed the estimation of the direct and indirect effects of hyperglycemia on mortality.

**Results:**

Glucose at admission ≥ 165 mg/dL and reduced renal function were associated with an increased risk of in-hospital mortality and length of hospitalization (all p < 0.001), while an increase in inflammatory parameters was significantly associated with an increased risk of all outcomes. High basophil count was associated with reduced mortality (p < 0.001). Hyperglycemia had a direct effect on mortality (p < 0.001); the indirect, through inflammatory markers, was significant only for absolute neutrophil count, C-Reactive protein and procalcitonin (p = 0.007, p = 0.029, p = 0.042). Patients with microvascular complications and with chronic kidney disease showed higher mortality (p = 0.03, p = 0.01).

**Conclusions:**

Hyperglycemia at admission, renal function and inflammatory parameters were found to be predictors of in-hospital mortality, while an increased basophil count was protective. Hyperglycemia had a direct effect on mortality, the indirect effect was only through few markers and markedly lower than the direct one.

## Introduction

The Coronavirus disease (COVID-19) has been a major cause of mortality worldwide because of severe acute respiratory syndrome [1] and abnormal systemic inflammatory response with endothelial damage [2–4].

Patients with hypertension, cancer, cardiovascular diseases, diabetes mellitus (DM), older age, and acute kidney injury, have a higher risk for developing more severe cases of COVID-19, as well as suffering a higher risk of mortality [5, 6]. Moreover, people with diabetes have a higher overall risk of infection resulting from hyperglycemia and multiple alterations of innate immunity [7–10].

One question of interest is the potential role of glucose control (past glycemic control, blood glucose at admission and during hospital stay) on outcomes related to COVID-19 in diabetic patients [11]. Holman et al*.* reported a higher risk of mortality from COVID-19 in patients with either type 1 or type 2 diabetes and glycated hemoglobin (HbA1c) of more than 10% compared with those with HbA1c of less than 6.5% [10]. Surprisingly, in the CORONADO study no significant association was noted between HbA1c concentrations and the primary composite outcome [12]. In the Veterans study a HbA1c ≥ 9.0%, was directly associated with adverse outcomes [13]. Finally, hyperglycemia has been found as an independent factor associated with severe prognosis in people hospitalized for COVID-19 [14–16].

How hyperglycemia is able to determine an adverse outcome is still under debate. DM is a well-established risk factor for COVID-19; however, the underlying mechanisms are unclear. On one hand, several studies indicated that the over-production of pro-inflammatory cytokines results in a so-called cytokine storm, which leads to high risk of vascular hyperpermeability, multiorgan failure, and death [2, 3]. High blood concentrations of inflammatory markers, a high neutrophil-to-lymphocyte ratio (N/L ratio), and increased blood concentrations of inflammatory cytokines and chemokines have been associated with both COVID-19 severity and death [2, 3]. On the other hand, hyperglycemia has been considered an independent predictor of mortality (16) and seems that hyperglycemia may affect COVID-19 related outcomes through noninflammatory processes [16].

The role of inflammation and hyperglycemia and their possible correlations has been investigated, trying to understand the interplay between inflammatory markers and hyperglycemia, a major factor in the development of multiorgan damage and mortality in diabetic patients with COVID-19 [16]; however, it is not clear whether there is a direct or indirect effect between these two factors.

Even if mortality is the most studied outcome, other outcomes such as in-hospital death, length of hospitalization, respiratory failure and need of intensive unit care are poorly described and possible predictors of all these outcomes together are almost unknown. It would be useful to identify which factors could predict the outcomes immediately at hospital admission, not only to complete a pathophysiological knowledge, but also to act on these in advance, improving patient outcomes.

The aim of this study was to identify predictors of COVID-19 related outcomes, as in-hospital mortality, length of hospitalization, respiratory failure and need of intensive unit care, considering the role of hyperglycemia, inflammation markers and clinical history.

## Research design and methods

### Study design and patients

A single-center retrospective observational study of a cohort of diabetic patients hospitalized with COVID-19 in Spedali Civili di Brescia (SCBH), the university hospital of one of the hardest hit cities in Europe, from February 1th 2020 to March 31th 2021, who also had an out-patient diabetic electronic record in the same hospital. A total of 291 patients with DM has been included. The study was approved from the Local Ethical Committee of SCBH.

### Data sources

An electronic health repository, called Diabsars, collected data of adults diabetic patients (≥ 18 years), identified by a unique alphanumeric code, hospitalized for COVID-19 in the SCBH, from February 1th 2020 to March 31th 2021, who also had a previous out-patient diabetic electronic record (FenixAmb^®^, by EL.CO.) in the same hospital. Pregnant women were excluded. Clinical criteria to define COVID-19 disease were: positive nasopharyngeal/oropharyngeal swab for SARS-Cov-2 or COVID-19 diagnosis.

Data collected during hospitalization, through electronic records FenixOE^®^ by EL.CO., were: medical history, demographics, all laboratory tests from the hospital admission (blood sample within the first 24 h) until discharge, inpatient medical therapy (insulin, oral antidiabetic drugs, immunosuppressive and antiretroviral drugs, antibiotics), hospitalization course, complications (cardiac, thrombotic, neurological, infectious) and clinical outcomes derived from codes of hospital discharge forms.

Data collected from the diabetic electronic repository were: DM types and duration, smoke habit, Body Mass Index (BMI kg/m^2^), microvascular (diabetic retinopathy, neuropathy and diabetic kidney disease) and macrovascular complications (stroke, myocardial infarction, chronic heart disease, arterial occlusive disease), diabetic foot, as well as other co-morbidities (hypertension, malignancies), laboratory tests recorded 1 year before hospitalization, antidiabetic therapy at home (insulin, biguanide, sulfonylureas, Dipeptidyl Peptidase IV -DDPIV- inhibitors, Glucagon like peptide-1 receptor agonist -GLP1RA-, sodium-glucose cotransporter 2 -SGLT2- inhibitors).

Diabetes mellitus was certified by a documented diagnosis in the diabetic electronic record FenixAmb^®^. The presence and the severity of respiratory failure has been collected from clinical records during hospitalization. The equation to calculate estimated glomerular filtration rate (eGFR) was that of the Chronic Kidney Disease Epidemiology Collaboration (commonly known as the CKD-EPI) [17]. On the basis of these measurements, chronic kidney disease is classified into six stages of estimated glomerular filtration rate (1, 2, 3A, 3B, 4, and 5), with stage 1 representing normal values and higher stages reflecting different degree of renal failure.

### Laboratory data

In all patients, the following biomarkers were retrieved from the database: white blood cell count (WBC), absolute neutrophils, lymphocytes, eosinophils, basophils and monocyte count, neutrophil-to-lymphocite ratio (NLR), platelets count (PTL), D-dimer, fibrinogen, lactate dehydrogenase (LDH), C-Reactive Protein (CRP), ferritin, procalcitonin (PCT), creatinine and eGFR. These blood tests were analyzed within 24 h after admission to the hospital, according to our internal COVID-19 diagnostic and therapeutic protocol [18]. In particular WBC count and HbA1c specimens were collected in peripheral blood sampling microtainer tube containing K2EDTA and analyzed using an automated blood analyzer (Coulter LH 750) for WBC count and a G8 HPLC analyzer (Tosoh bioscience Inc) for HbA1c. The laboratory tests for fibrinogen and D-dimer were performed on blood collected with sodium citrate on ACL TOP (Instrumentation Laboratory, Milan, Italy), according to the manufacturer’s specifications and using HemosIL reagent system17 (Instrumentation Laboratory). To determine Glucose, PCR, LDH, PCT, ferritin and creatinine’s plasma levels blood was collected in lithium-heparin tube and commercially available assays were used according to manufacturer’s instruction (Roche diagnostic) within 1 h from collection.

### Outcomes

The outcomes of these study were: 1. in-hospital death, defined by codes of hospital discharge forms; 2. length of hospitalization; 3. presence of respiratory failure and need of oxygen therapy (level 1 only oxygen supplementation, level 2 mechanical ventilation, level 3 invasive mechanical ventilation); 4. intensive care unit admission, derived by codes of hospital discharge forms.

### Statistical analyses

The distributions of continuous variables were summarized by median and interquartile range (IQR), while for categorical variables the number of observations with percentages (%) were reported. Differences between median values and proportions in subgroups of participants were tested using Wilcoxon rank-sum and Fisher’s exact test, respectively.

The association between in-hospital mortality and covariates (laboratory parameters, inflammatory markers, indicators of renal function) were investigated using multivariable Cox regression. Each quantitative covariate was first split into 5 categories defined by quintiles and added to a multivariable model together with sex, age and BMI; four adjusted hazard ratios (HRs) were estimated and the significance of each HR was tested using a Wald test. Categorizing the covariates using quintiles can reveal the presence of discontinuous threshold associations or other nonlinear associations.

The relationships between covariates and hospital length of stay were analysed using a competing risk model as suggested by Brock et al. [19]; hospital discharge was the primary event and in-hospital death was treated as a competing event. A multivariable Fine and Gray regression model was fitted for each categorized covariate and adjusted subdistribution hazard ratios (SHR) were estimated. Values of SHR below 1 indicates an increase in length of stay in the hospital.

A multivariable logistic regression was used to model the associations between covariates and respiratory failure (a binary outcome), while the need of oxygen therapy (an ordinal response variable) was analysed by ordinal logistic regression. In the ordinal logistic case, the estimated odds ratio (OR) represents the variation, given a one-unit increase in the covariate, of the odds of being in a higher rather than a lower category [20].

The interplay among levels of glycemia (above/below 165 mg/dL), inflammation and in-hospital mortality has been investigated using a causal mediation analysis. The average direct effect (ADE) and the average causal mediation effect (ACME) of glycemia, mediated by inflammatory markers, were estimated using the method described by Tingley et al. [21]. For each marker, linear regression and parametric survival regression (with log-logistic distribution) were used for the mediator and outcome models, respectively. Sex, age, and BMI were considered in these models as potential confounders. ADE and ACME can be interpreted as differences in estimated median survival time between groups with levels of glycemia above and below the threshold of 165 mg/dL.

The statistical analyses were performed using Stata version 16.1 (Stata Corp., College Station, TX, USA) and R version 4.1.3 (R Foundation for Statistical Computing, Vienna, Austria). Statistical significance was set at p < 0.05.

## Results

### Study cohort characteristics

The overall cohort (n = 291) had a median age of 72 (63–79) years, 72.2% were men. All patients met the criteria for DM, of whom 96% was type 2, 7% type 1, and 7% secondary diabetes. Previous comorbidities were hypertension (43.3%), chronic kidney disease (42.2%), coronary artery disease (27.4%), diabetic foot (8.9%), retinopathy (17.8%), neuropathy (6.9%).

Laboratory data and inflammatory markers has been evaluated at admission and the therapy during hospitalization has been recorded. Chronic kidney disease (CKD) was defined by the presence of an eGFR, calculated by the CKD-EPI equation, of < 60 ml/min/m^2^. Demographic and clinical characteristics of patients are shown in Table 1.

**Table 1:** Demographic and clinical characteristics of patients at admission

Variable	Overall cohort (n = 291)
Age (years)	72 (63–79)
Male sex	210 (72.2%)
Type of diabetes	
Type 2	277 (95.2%)
Type 1	7 (2.4%)
Secondary diabetes	7 (2.4%)
Diabetes duration (years)	16 (1–51)
BMI (kg/m^2^)	28.6 (26.3–32.9)
History of tobacco use	93 (31.9%)
Comorbidities	
Hypertension	126 (43.3%)
Chronic kidney disease	122 (42.2%)
Coronary artery disease	80 (27.4%)
Diabetic foot	26 (8.9%)
Retinopathy	52 (17.8%)
Neuropathy	20 (6.9%)
Malignancy	20 (6.9%)
Laboratory data	
Glucose (mg/dl)	152.5 (108–199)
HbA1c (mmol/mol)	66 (58–79)
HbA1c (%)	8.2 (7.5–9.4)
Platelets count (PLT 10^3^/μL)	229,500 (161,000–331,000)
White blood cell count (WBC 10^3^/μL l)	7.32 (5.53–10.22)
Absolute basophil count (10^3^/μL)	0.02 (0.43–0.84)
Absolute lymphocyte count (10^3^/ μL)	1.22 (0.75–1.68)
Absolute eosinophils count (10^3^/μL)	0.08 (0.010–0.17)
Absolute neutrophil count (10^3^/μL)	4.98 (3.41–8.11)
Absolute monocyte count (10^3^/μL)	8.4 (5.3–10.5)
CRP (mg/l)	16 (4.3–56.8)
Fibrinogen (mg/dl)	443 (335–558)
D-Dimer (ng/ml)	514 (294–1208)
Ferritin (μg/l)	594 (291–1087)
Procalcitonin (ng/ml)	0.20 (0.10–0.49)
NLR	4.06 (2.25–8.92)
LDH (U/l)	276 (223–365)
Creatinine (mg/dl)	1.04 (0.8–1.6)
eGFR CDK-EPI (ml/min × 1.73 m^2^)	
≤ 30	48 (16.6%)
30–75	112 (38.7%)
> 75	129 (44.6%)
Outcomes	
In hospital death	74 (25%)
Need of oxygen supplementation	151 (51.9%)
Intensive care unit	20 (6.9%)
Length of hospitalization (days)	14 (8–23)
Therapy during hospitalization	
Steoroids	151 (57.4%)
Hydroxyclorochine	149 (56.6%)
Antiretrovirals	148 (56.2%)
Tocilizumab/kanakinumab	17 (6.6%)
Antibiotics	216 (82.4%)
Insulin	142 (53.9%)

Continuous variables are summarized as median and interquartile range (IQR); categorical variables as number and percentage of patients

*BMI* Body Mass Index, *HbA1c* Glycated Haemoglobin, *CRP* C-Reactive Protein, *NLR* Neutrophil-to-Lymphocyte Ratio, *LDH* Lactate Dehydrogenase, *eGFR* Estimated Glomerular Filtration Rate, *CDK-EPI* Chronic Kidney Disease Epidemiology Collaboration

Table 2 shows the results of the association analysis between outcomes and potential predictors.

**Table 2:** Predictors of outcomes in diabetic patients hospitalized for COVID-19

		Mortality		Length of hospitalization		Respiratory failure		Need of oxygen therapy	
Variable	Interval (by quintiles)	HR (95%CI)	P	SHR (95%CI)	P	OR (95%CI)	P	OR (95%CI)	P
Glucose (mg/dL)	56–102	Reference	–	Reference	–	Reference	–	Reference	–
	103–133	1.70 (0.62 – 4.70)	0.3	0.73 (0.48 – 1.13)	0.2	0.98 (0.43 – 2.27)	1	1.00 (0.44 – 2.23)	0.9
	134–164	1.64 (0.60 – 4.49)	0.3	0.72 (0.46 – 1.11)	0.1	1.81 (0.77 – 4.27)	0.2	1.92 (0.89 – 4.15)	0.1
	165–220	3.86 (1.50 – 9.92)	**0.005**	0.70 (0.43 – 1.12)	0.1	1.44 (0.62 – 3.36)	0.4	1.26 (0.59 – 2.69)	0.5
	221–452	4.71 (1.87 – 11.9)	**0.001**	0.53 (0.33 – 0.84)	**0.008**	1.63 (0.70 – 3.82)	0.3	1.26 (0.59 – 2.71)	0.5
WBC (10^3^/μL)	1.97–5.22	Reference	–	Reference	–	Reference	–	Reference	–
	5.23–6.54	1.34 (0.45 – 4.01)	0.6	1.16 (0.79 – 1.70)	0.2	0.70 (0.32 – 1.52)	0.4	0.80 (0.38 – 1.68)	0.6
	6.55–8.10	1.68 (0.58 – 4.89)	0.3	0.85 (0.57 – 1.28)	0.8	0.83 (0.38 – 1.83)	0.6	0.93 (0.45 – 1.91)	0.8
	8.11–11.10	2.50 (0.96 – 6.49)	0.059	0.65 (0.44 – 0.96)	**0.031**	1.50 (0.66 – 3.41)	0.3	1.58 (0.77 – 3.25)	0.2
	11.11–45.18	7.11 (2.96 – 17.1)	** < 0.001**	0.29 (0.17 – 0.49)	** < 0.001**	2.08 (0.87 – 4.99)	0.1	1.76 (0.85 – 3.64)	0.1
Absolute neutrophil count (10^3^/μL)	0.45–3.12	Reference	–	Reference	–	Reference	–	Reference	–
	3.13–4.35	6.64 (0.80 – 55.4)	0.08	0.89 (0.63 – 1.26)	0.5	0.68 (0.31 – 1.50)	0.3	0.80 (0.37 – 1.70)	0.6
	4.36–5.92	6.97 (0.81 – 60.1)	0.08	0.96 (0.67 – 1.36)	0.8	0.85 (0.39 – 1.86)	0.7	1.36 (0.63 – 2.55)	0.4
	5.93–9.08	25.0 (3.31 – 188.2)	**0.002**	0.48 (0.32 – 0.72)	** < 0.001**	1.33 (0.59 – 3.02)	0.5	1.59 (0.85 – 3.41)	0.2
	9.09–42.74	50.1 (6.80 – 369.5)	** < 0.001**	0.21 (0.12 – 0.38)	** < 0.001**	2.34 (0.95 – 5.75)	0.065	2.12 (0.98 – 4.01)	**0.048**
Absolute lymphocyte count (10^3^/μL)	0.09–0.66	Reference	–	Reference	–	Reference	–	Reference	–
	0.67–0.98	0.53 (0.30 – 0.94)	**0.03**	2.38 (1.30 – 4.35)	**0.005**	0.65 (0.27 – 1.60)	0.4	0.64 (0.31 – 1.32)	0.2
	0.99–1.43	0.22 (0.09 – 0.50)	** < 0.001**	4.11 (2.31 – 7.31)	** < 0.001**	0.32 (0.13 – 0.76)	**0.010**	0.46 (0.22 – 0.97)	**0.040**
	1.44–1.79	0.09 (0.03 – 0.29)	** < 0.001**	4.15 (2.39 – 7.21)	** < 0.001**	0.48 (0.20 – 1.18)	0.1	0.49 (0.23 – 1.03)	0.061
	1.80–6.45	0.08 (0.02 – 0.27)	** < 0.001**	3.61 (2.09 – 6.25)	** < 0.001**	0.39 (0.16 – 0.95)	**0.039**	0.35 (0.16 – 0.76)	**0.008**
Absolute monocyte count (10^3^/μL)	0.10–0.38	Reference	–	Reference	–	Reference	–	Reference	–
	0.39–0.50	0.54 (0.27 – 1.11)	0.09	2.22 (1.37 – 3.60)	**0.001**	0.34 (0.15 – 0.81)	**0.014**	0.36 (0.17 – 0.75)	**0.006**
	0.51–0.64	0.47 (0.23 – 0.98)	**0.044**	1.92 (1.22 – 3.03)	**0.005**	0.49 (0.20 – 1.15)	0.1	0.58 (0.29 – 1.16)	0.1
	0.65–0.82	0.25 (0.09 – 0.65)	**0.005**	2.65 (1.70 – 4.14)	** < 0.001**	0.28 (0.12 – 0.65)	**0.003**	0.41 (0.20 – 0.85)	**0.017**
	0.83–1.89	0.63 (0.32 – 1.24)	0.2	1.63 (1.03 – 2.57)	**0.035**	0.61 (0.25 – 1.48)	0.3	0.43 (0.21 – 0.90)	**0.024**
Absolute eosinophil count (10^3^/μL)	0.00–0.00	Reference	–	Reference	–	Reference	–	Reference	–
	0.01–0.04	0.51 (0.27 – 0.95)	**0.034**	2.10 (1.18 – 3.74)	**0.011**	0.67 (0.27 – 1.68)	0.4	0.75 (0.36 – 1.54)	0.4
	0.05–0.12	0.10 (0.04 – 0.25)	** < 0.001**	2.84 (1.58 – 5.14)	**0.001**	0.37 (0.16 – 0.86)	**0.021**	0.65 (0.32 – 1.29)	0.2
	0.12–0.20	0.11 (0.04 – 0.28)	** < 0.001**	3.75 (2.23 – 6.30)	** < 0.001**	0.45 (0.19 – 1.08)	0.072	0.80 (0.40 – 1.61)	0.5
	0.21–1.71	0.06 (0.02 – 0.18)	** < 0.001**	3.06 (1.85 – 5.05)	** < 0.001**	0.25 (0.10 – 0.59)	**0.002**	0.36 (0.17 – 0.80)	**0.008**
Absolute basophil count (10^3^/μL)	0.00–0.01	Reference	–	Reference	–	Reference	–	Reference	–
	0.02–0.02	0.99 (0.51 – 1.92)	1	1.02 (0.64 – 1.64)	0.9	0.38 (0.18 – 0.82)	**0.014**	0.59 (0.30 – 1.19)	0.1
	0.03–0.03	0.21 (0.07 – 0.59)	**0.003**	1.48 (1.02 – 2.14)	**0.037**	0.43 (0.20 – 0.92)	**0.030**	0.54 (0.27 – 1.06)	0.07
	0.04–0.05	0.56 (0.27 – 1.20)	0.1	1.03 (0.69 – 1.55)	0.9	0.54 (0.25 – 1.17)	0.1	0.84 (0.43 – 1.64)	0.6
	0.06–0.18	0.44 (0.20 – 0.96)	**0.038**	0.84 (0.55 – 1.28)	0.4	0.51 (0.22 – 1.19)	0.1	0.66 (0.31 – 1.42)	0.3
NLR †	0.40–1.83	NE	NE	Reference	–	Reference	–	Reference	–
	1.84–3.23	0.02 (0.003 – 0.15)	** < 0.001**	1.15 (0.85 – 1.54)	0.4	0.56 (0.25 – 1.24)	0.2	0.66 (0.31 – 1.39)	0.3
	3.24–5.46	0.11 (0.04 – 0.31)	** < 0.001**	1.05 (0.75 – 1.47)	0.8	0.60 (0.27 – 1.35)	0.2	0.75 (0.36 – 1.57)	0.4
	5.47–11.10	0.64 (0.35 – 1.17)	0.1	0.52 (0.33 – 0.81)	**0.005**	0.99 (0.43 – 2.27)	0.9	1.31 (0.62 – 2.77)	0.5
	11.11–97.14	Reference^†^	–	0.14 (0.07 – 0.25)	** < 0.001**	4.13 (1.45 – 11.8)	**0.008**	2.49 (1.18 – 5.23)	**0.016**
PLT (10^3^/μL)	20–149	Reference	–	Reference	–	Reference	–	Reference	–
	150–198	1.56 (0.85 – 2.87)	0.1	0.93 (0.57 – 1.54)	0.8	0.82 (0.35 – 1.90)	0.6	0.62 (0.30 – 1.30)	0.2
	199–270	0.63 (0.30 – 1.31)	0.2	1.82 (1.16 – 2.86)	**0.009**	1.05 (0.44 – 2.50)	0.9	0.48 (0.23 – 1.01)	0.052
	271–350	0.44 (0.19 – 1.04)	0.062	2.05 (1.31 – 3.20)	**0.002**	0.56 (0.24 – 1.28)	0.2	0.51 (0.25 – 1.06)	0.073
	351–747	0.39 (0.16 – 0.97)	**0.043**	2.50 (1.59 – 3.95)	** < 0.001**	0.53 (0.23 – 1.22)	0.1	0.42 (0.20 – 0.87)	**0.020**
D–Dimer (ng/mL)	200–250	Reference	–	Reference	–	Reference	–	Reference	–
	251–415	2.27 (0.25 – 20.7)	0.5	0.56 (0.32 – 0.97)	**0.040**	2.01 (0.67 – 6.01)	0.2	1.20 (0.41 – 3.61)	0.7
	416–665	2.27 (0.26 – 19.7)	0.5	0.52 (0.29 – 0.92)	**0.025**	10.4 (2.55 – 42.5)	**0.001**	3.77 (1.25 – 11.4)	**0.018**
	666–2467	4.06 (0.50 – 33.1)	0.2	0.41 (0.23 – 0.75)	**0.004**	4.46 (1.36 – 14.6)	**0.013**	5.02 (1.66 – 15.2)	**0.004**
	2468–16781	6.94 (0.89 – 54.2)	0.065	0.26 (0.12 – 0.56)	**0.001**	3.50 (1.01 – 12.2)	**0.048**	2.01 (0.62 – 6.54)	0.2
Fibrinogen (mg/dL)	105–303	Reference	–	Reference	–	Reference	–	Reference	–
	304–405	2.20 (0.77 – 6.29)	0.1	1.21 (0.79 – 1.88)	0.4	1.20 (0.47 – 3.07)	0.7	0.95 (0.41 – 2.20)	0.9
	406–487	1.33 (0.48 – 3.72)	0.6	1.06 (0.70 – 1.61)	0.8	0.72 (0.30 – 1.75)	0.5	0.47 (0.20 – 1.08)	0.075
	488–596	1.10 (0.35 – 3.43)	0.9	1.39 (0.92 – 2.09)	0.1	1.03 (0.42 – 2.55)	0.9	0.62 (0.27 – 1.40)	0.2
	597–1113	3.12 (1.22 – 7.92)	**0.017**	0.66 (0.38 – 1.12)	0.1	3.66 (1.23 – 10.9)	**0.020**	1.41 (0.63 – 3.17)	0.4
CRP (mg/L)	0.3–3.4	Reference	–	Reference	–	Reference	–	Reference	–
	3.4–9.3	0.86 (0.05 – 13.8)	0.9	1.11 (0.82 – 1.49)	0.5	0.65 (0.30 – 1.43)	0.3	0.51 (0.24 – 1.09)	0.08
	9.3–25.7	6.81 (0.85 – 54.8)	0.072	0.74 (0.52 – 1.04)	0.08	1.55 (0.68 – 3.54)	0.3	0.93 (0.45 – 1.89)	0.8
	25.7–78.6	18.4 (2.42 – 140.3)	**0.005**	0.69 (0.44 – 1.07)	0.1	1.16 (0.51 – 2.61)	0.7	0.87 (0.42 – 1.83)	0.7
	78.6–435.4	53.4 (7.19 – 397.1)	** < 0.001**	0.12 (0.06 – 0.23)	** < 0.001**	2.56 (1.02 – 6.40)	**0.045**	2.10 (0.99 – 4.47)	0.053
PCT (ng/mL)	0.10–0.10	Reference	–	Reference	–	Reference	–	Reference	–
	0.11–0.12	NE	NE	2.61 (1.52 – 4.48)	**0.001**	1.18 (0.20 – 6.95)	0.9	3.09 (0.74 – 12.9)	0.1
	0.12 – 0.30	1.61 (0.52 – 4.97)	0.4	0.83 (0.48 – 1.43)	0.5	0.77 (0.28 – 2.11)	0.6	1.28 (0.55 – 2.99)	0.6
	0.31 – 0.62	3.43 (1.28 – 9.20)	**0.014**	0.45 (0.24 – 0.83)	**0.011**	2.91 (0.83 – 10.2)	0.1	2.62 (1.11 – 6.18)	**0.028**
	0.63 – 21.20	3.42 (1.27 – 9.18)	**0.015**	0.39 (0.21 – 0.72)	**0.002**	1.52 (0.50 – 4.57)	0.5	3.15 (1.33 – 7.47)	**0.009**
Ferritin (μg/L)	18 – 241	Reference	–	Reference	–	Reference	–	Reference	–
	242 – 449	3.11 (0.85 – 11.3)	0.085	0.76 (0.50 – 1.16)	0.2	1.33 (0.55 – 3.25)	0.6	1.40 (0.62 – 3.20)	0.4
	450 – 680	4.19 (1.15 – 15.2)	**0.029**	0.80 (0.53 – 1.22)	0.3	1.75 (0.73 – 4.20)	0.2	1.73 (0.78 – 3.84)	0.2
	681 – 1285	4.59 (1.29 – 16.3)	**0.018**	0.64 (0.42 – 0.98)	**0.041**	2.44 (0.97 – 6.13)	0.058	2.65 (1.20 – 5.87)	**0.016**
	1286 – 10477	5.22 (1.45 – 18.8)	**0.011**	0.69 (0.42 – 1.13)	0.1	1.90 (0.75 – 4.82)	0.2	1.66 (0.73 – 3.78)	0.2
LDH (U/L)	136 – 213	Reference	–	Reference	–	Reference	–	Reference	–
	214 – 250	0.40 (0.08 – 2.08)	0.3	0.97 (0.63 – 1.47)	0.9	1.06 (0.47 – 2.41)	0.9	0.80 (0.37 – 1.72)	0.6
	251 – 299	1.32 (0.43 – 4.08)	0.6	0.79 (0.51 – 1.22)	0.3	1.33 (0.59 – 2.98)	0.5	0.98 (0.46 – 2.09)	1
	300 – 391	1.97 (0.69 – 5.63)	0.2	0.59 (0.37 – 0.94)	**0.026**	1.43 (0.63 – 3.26)	0.4	1.25 (0.58 – 2.71)	0.6
	392 – 1092	5.20 (2.00 – 13.5)	**0.001**	0.19 (0.10 – 0.35)	** < 0.001**	8.96 (2.75 – 29.2)	** < 0.001**	2.68 (1.25 – 5.74)	**0.011**
Creatinine (mg/dL)	0.39 – 0.76	Reference	–	Reference	–	Reference	–	Reference	–
	0.77 – 0.94	0.78 (0.26 – 2.33)	0.7	1.23 (0.83 – 1.81)	0.3	1.30 (0.56 – 3.02)	0.5	0.94 (0.45 – 1.96)	0.9
	0.95–1.23	1.18 (0.44 – 3.21)	0.7	1.24 (0.82 – 1.89)	0.3	0.73 (0.32 – 1.67)	0.5	0.87 (0.40 – 1.87)	0.7
	1.24–1.92	1.78 (0.72 – 4.38)	0.2	0.79 (0.51 – 1.22)	0.3	1.10 (0.48 – 2.51)	0.8	0.72 (0.36 – 1.53)	0.4
	1.92–11.96	3.23 (1.38 – 7.60)	**0.007**	0.42 (0.25 – 0.72)	**0.002**	1.27 (0.53 – 3.04)	0.6	1.13 (0.52 – 2.45)	0.8
eGFR CDK–EPI (ml/min × 1.73 m2)	3.3–32.9	Reference	–	Reference	–	Reference	–	Reference	–
	33.0–57.9	0.37 (0.19–0.73)	**0.004**	2.16 (1.30 – 3.57)	**0.003**	0.75 (0.34 – 1.65)	0.5	0.59 (0.29 – 1.21)	0.1
	58.0–80.9	0.63 (0.34–1.16)	0.1	1.76 (1.02 – 3.03)	**0.041**	0.75 (0.33 – 1.68)	0.5	0.96 (0.47 – 1.97)	0.9
	81.0–96.1	0.21 (0.09–0.52)	**0.001**	3.07 (1.86 – 5.06)	** < 0.001**	0.86 (0.38 – 1.94)	0.7	0.64 (0.31 – 1.30)	0.2
	96.2–126.6	0.22 (0.07–0.65)	**0.006**	2.34 (1.39–3.94)	**0.001**	0.95 (0.38 – 2.40)	0.9	1.28 (0.56 – 2.88)	0.6

Bold values indicate statistically significant results, where *p* < 0.05

*HR* hazard ratio, *SHR* subhazard ratio, *OR* odds ratio, *CI* confidence interval, *P* p-value of the Wald test on the model coefficient, *WBC* White Blood Cell Count, *NLR* Neutrophil-to-Lymphocyte Ratio, *PLT* Platelet Count, *CRP* C-Reactive Protein, *PCT* Procalcitonin, *LDH* Lactate Dehydrogenase, *eGFR* Estimated Glomerular Filtration Rate, *CDK-EPI* Chronic Kidney Disease Epidemiology Collaboration

*NE* the effect was not estimable,

HR, SHR e OR have been adjusted by sex, age and BMI;

Need of oxygen therapy: level 1 only oxygen supplementation, level 2 mechanical ventilation, level 3 invasive mechanical ventilation;

Presence of Respiratory failure: yes, no

All the variables were measured at admission

^†^ The reference class for the association between NLR and mortality was set to the last quintile (in the first quintile no events occurred);

### Predictors of Mortality (in-hospital death)

Significantly increased mortality was observed for glucose ≥ 165 mg/dL (p < 0.001), white blood cell (WBC) count ≥ 11.11 10^3^/μL, (p < 0.001), absolute neutrophil count ≥ 5.93 10^3^/μL (p < 0.001), absolute eosinophil count < 0.01 10^3^/μL (p < 0.001), absolute lymphocyte count < 0.99 10^3^/μL (p < 0.001), neutrophil-to-lymphocyte ratio (NLR) ≥ 5.47 (p < 0.001), platelet count (PLT) ≤ 270 10^3^/μL (p = 0.003), fibrinogen ≥ 597 mg/dL (p = 0.004), procalcitonin (PCT) > 0.3 ng/mL (p = 0.002), ferritin > 241 μg/L (p = 0.018), lactate dehydrogenase (LDH) ≥ 392 U/L (p < 0.001), and creatinine ≥ 1.23 mg/dL (p < 0.001).

Significantly reduced mortality was evidenced for absolute basophil count ≥ 0.02 103/μL (p < 0.001).

C-reactive protein (CRP) showed a linear positive association with mortality (HR = 1.01, 95%CI 1.008–1.012; p < 0.001), without a cut off, as previous variables. eGFR CDK–EPI showed a linear negative association with mortality (HR = 0.984, 95%CI 0.976–0.992; p < 0.001), without a cut off as previous variables.

When considering complications during hospitalization, only respiratory failure complications were significantly associated with an increased mortality (HR = 2.9, 95%CI 1.5–5.9; p = 0.003). Infectious, cardiovascular, thromboembolic or neurological complications did not show a significant association with mortality.

**Fig. 1:**
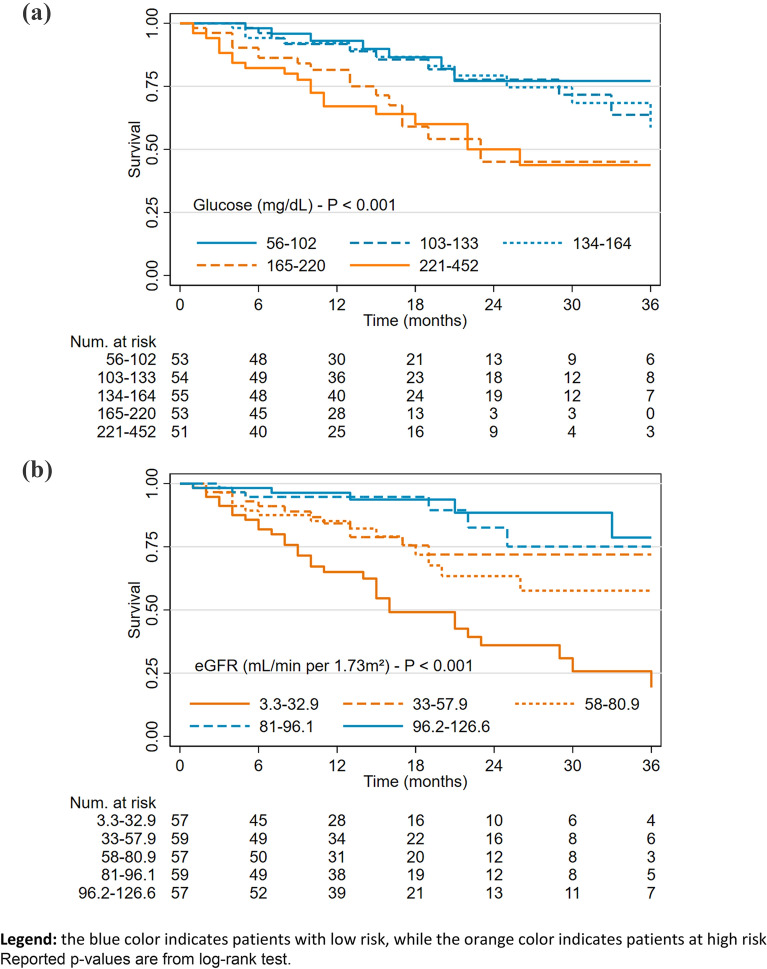
Kaplan–Meier curves depicting the association between in-hospital mortality and glucose levels (**a**) and renal function at admission (**b**)

Kaplan–Meier curves were used to depict the association between mortality and glucose at admission (Fig. 1a), and between mortality and renal function (Fig. 1b).

The mediation analysis evidenced that the direct effect of glucose on mortality was statistically significant. By the contrary, the indirect effect of glucose, mediated by inflammatory markers, was significant only for absolute neutrophil count, CRP and PCT (p = 0.007, p = 0.029, p = 0.042, respectively); these results suggest that glucose might have an indirect effect on mortality only through a small set of markers and this indirect effect was markedly lower than the direct one.

The results are summarized in Table 3.

**Table 3:** Explorative mediation analysis of the direct and indirect effects of glucose on mortality; the average causal mediation effect (ACME) and the average direct effect (ADE) represent the estimated averages of the mediation and direct effects, respectively

Mediator	ACME (95% CI)	P	ADE (95% CI)	P
WBC (× 10^3^/μL)	− 5.3 (− 14.8 to 0.10)	0.054	− 45.7 (− 90.4 to − 15.9)	0.001
Absolute neutrophil count (× 10^3^/μL)	− 10.0 (− 24.6 to − 1.6)	0.007	− 31.8 (− 63.3 to − 6.5)	0.011
Absolute lymphocyte count (× 10^3^/μL)	− 8.6 (− 36.1 to 9.8)	0.4	− 50.5 (− 101.0 to − 17.4)	0.003
Absolute monocyte count (× 10^3^/μL)	0.0 (− 1.9 to 2)	1	− 37.6 (− 69.1 to − 15.0)	0.001
Absolute eosinophil count (× 10^3^/μL)	− 23.1 (− 98.3 to 2.4)	0.1	− 50.0 (− 123.4 to − 15.7)	0.004
Absolute basophil count (× 10^3^/μL)	0.7 (− 3.2 to 5.8)	0.8	− 39.5 (− 74.3 to − 15.6)	< 0.001
NLR	− 4.1 (− 13.3 to 2.1)	0.2	− 39.0 (− 73.7 to − 13.7)	0.001
PLT (× 10^3^/μL)	0.1 (− 4.7 to 5.3)	1	− 48.1 (− 91.3 to − 19.1)	< 0.001
D–Dimer (ng/mL)	− 0.3 (− 5.8 to 4.3)	0.9	− 41.5 (− 97.5 to − 4.7)	0.022
Fibrinogen (mg/dL)	− 1.2 (− 6.2 to 2.2)	0.5	− 40.0 (− 81.3 to − 12.3)	0.002
CRP (mg/L)	− 9.4 (− 21.6 to − 0.9)	0.029	− 40.4 (− 74.6 to − 16.2)	< 0.001
PCT (ng/mL)	− 1.8 (− 4.7 to 0.0)	0.042	− 27.0 (− 53.0 to − 6.3)	0.009
Ferritin (μg/L)	− 1.6 (− 6.6 to 1.3)	0.3	− 31.3 (− 57.5 to − 12.0)	< 0.001
LDH (U/L)	− 5.8 (− 14.7 to 0.9)	0.09	− 30.6 (− 62.3 to − 8.3)	0.003
Creatinine (mg/dL)	− 0.3 (− 3.2 to 2.3)	0.8	− 43.0 (− 80.8 to − 16.5)	< 0.001
eGFR–CDK-EPI (ml/min × 1.73 m2)	1.0 (− 4.1 to 6.9)	0.7	− 48.5 (− 94.3 to − 18.3)	< 0.001

*CI* confidence interval, *P* p-value of the test for the average indirect and direct effects, *WBC* White Blood Cell Count, *NLR* Neutrophil-to-Lymphocyte Ratio, *PLT* Platelet Count, *CRP* C-Reactive Protein, *PCT* Procalcitonin, *LDH* Lactate Dehydrogenase, *eGFR* Estimated Glomerular Filtration Rate, *CDK-EPI* Chronic Kidney Disease Epidemiology Collaboration

### Predictors of length of hospitalization

WBC count ≥ 8.11 10^3^/μL (p < 0.001), absolute neutrophil count ≥ 5.93 10^3^/μL (p < 0.001), absolute lymphocyte count < 0.67 10^3^/μL (p < 0.001), absolute monocyte count < 0.39 10^3^/μL (p < 0.001), absolute eosinophil count < 0.01 10^3^/μL (p < 0.001), NLR ≥ 5.47 (p < 0.001), PLT < 199 10^3^/μL (p < 0.001), D-Dimer > 250 ng/mL (p = 0.003), CRP ≥ 78.6 mg/L (p < 0.001), LDH ≥ 300 U/L (p < 0.001), creatinine ≥ 1.93 mg/dL (p < 0.001), eGFR CDK–EPI < 33 ml/min × 1.73 m^2^ (p < 0.001) were found associated with a significantly longer length of hospitalization.

Glucose levels at admission had a positive linear association with length of hospitalization (SHR = 0.997, 95%CI 0.995–0.999; p = 0.007).

More details of this analysis can be found in Table 2*.*

### Predictors of respiratory failure

Higher rates of respiratory failure were observed for WBC count ≥ 8.11 10^3^/μL (p = 0.007), absolute neutrophil count ≥ 5.93 10^3^/μL (p = 0.009), absolute lymphocyte count < 0.99 10^3^/μL (p = 0.012), absolute monocyte count < 0.39 10^3^/μL (p = 0.004), absolute eosinophil count < 0.05 10^3^/μL (p = 0.002), NLR ≥ 11.11 (p < 0.001), D-Dimer ≥ 416 ng/mL (p = 0.002), Fibrinogen ≥ 597 mg/dL (p = 0.005), C-Reactive Protein ≥ 78.6 mg/L (p = 0.014), LDH ≥ 392 U/L (p < 0.001). Glucose levels, creatinine and eGFR values were not found significantly associated with respiratory failure.

Absolute neutrophil count ≥ 9.09 10^3^/μL (p = 0.045), absolute lymphocyte count < 0.99 10^3^/μL (p = 0.015), absolute monocyte count ≥ 0.39 10^3^/μL, absolute eosinophil count < 0.21 10^3^/μL (p = 0.016), NLR ≥ 11.11 (p = 0.001), PLT < 199 10^3^/μL (p = 0.033), D-Dimer ≥ 416 ng/mL (p = 0.002), C-Reactive Protein ≥ 78.6 mg/L (p = 0.001), PCT > 0.3 ng/mL (p = 0.007), and LDH ≥ 392 U/L (p = 0.001) were significantly associated with an increased need of oxygen therapy. No significant association was found for glucose levels, creatinine and eGFR values. The results are summarized in Table 2*.*

### Predictors of intensive care unit admission

The following markers were found associated with an increased risk of intensive care unit (ICU) admission: absolute lymphocyte count (OR = 0.33, 95%CI 0.13–0.80; p = 0.015), neutrophil-to-lymphocyte ratio (OR = 1.04, 95%CI 1.01–1.07; p = 0.017), platelet count (OR = 0.994, 95%CI 0.989– 0.999; p = 0.014), C-reactive protein (OR = 1.008, 95%CI 1.003–1.012; p = 0.001). Higher rates of ICU admission were also found in patients with levels of eGFR CDK–EPI ≤ 75 ml/min × 1.73 m^2^ (OR = 3.7, 95%CI 1.2–11.3; p = 0.022) and with creatinine levels ≥ 2 mg/dL (OR = 3.1, 95%CI 1.1–8.8; p = 0.032).

Glucose levels and ICU did not show a statistically significant association.

### Pre-hospital status and clinical outcomes during hospitalization

Patients with microvascular complications and chronic kidney disease showed higher mortality rates during hospitalization (p = 0.03 and p = 0.01, respectively). No association of the other variables before hospitalization with clinical outcomes has been evidenced.

The patients treated with metformin before hospitalization had a reduced risk of mortality during hospitalization (p = 0.016). No association of the other medication before hospitalization with clinical outcomes has been evidenced.

No relationship between HbA1c, duration of diabetes and the outcomes of the study has been found.

## Discussion

In this study we found potential predictors of multiple and complex clinical outcomes, considering the role of hyperglycemia, inflammation markers and clinical history. This information allowed us to add evidence to what has been debated or hypothesized in the literature.

High level of glucose at admission, an increase in inflammatory parameters and impaired renal function at admission to the hospital were found associated with an increased mortality during hospitalization, independently from BMI, age and sex. The mean age of our population was 72 years, one would expect that age alone would be responsible for the increased mortality as reported in literature [12]; however, hyperglycemia, inflammation and impaired renal function seems to be important, independently of age. Moreover, in this population even a slightly higher glucose level as 165 mg/dL, that may be tolerate in daily management, was associated with an increased mortality. This is an intriguing point; we can speculate that in older people the cut off level of hyperglycemia leading to an increased risk of mortality during COVID-19 might be lower than in younger; therefore, an early check and control of glycemia is important.

The full inflammatory profile in DM patients with COVID-19 is not characterized yet, however, hyperinflammation could be a possible response to the infection. Indeed, cytokine storm, an activation cascade of auto-amplifying cytokines production due to unregulated host immune response to COVID-19 infection, has been proposed as a pathological mechanism [22].

Diabetes is also characterized by chronic, low-grade inflammation, which is a prominent feature of its complications, and its pathophysiology shares several proinflammatory molecules from the COVID-19 cytokine storm cascade [23, 24]. The underlying chronic inflammatory state in diabetes may be “locked and loaded” for virus-induced damage, promoting a vicious cycle of cytokine release, leading to more widespread multiorgan damage, including injury to tissues already weakened by pre-existing diabetes complications [25]. Different authors have hypothesized that, as a chronic inflammatory condition, DM may predispose individuals to an increased inflammatory response since hyperglycaemia has traditionally been thought to be a major driver of inflammation [26]. However, in our study, the indirect effect of glucose on mortality through inflammatory markers was not significant for the majority of inflammatory biomarkers evaluated, except for absolute neutrophil count, CRP and PCT. Finally, hyperglycemia at admission to the hospital had a direct effect, not mediated by inflammation, on mortality.

Our data confirm what was recently described by Kho et al. which hypothesized that CRP is a partial mediator of the association between DM and severe COVID-19 [27], while, to our knowledge, we firstly described a similar role for PCT. It is known that PCT is associated with insulin resistance and association of plasma PCT in the general population [23], and now it has also a similar feature in diabetic COVID-19 patient.

This is an intriguing point because CRP and PCT could have a double effect on mortality, both direct and indirect mediated by hyperglycemia, being key markers and predictors of this outcome. Inflammation alone is also responsible of an increased mortality, probably mediated by the COVID-19 cytokine storm cascade. Therefore, both inflammation and hyperglycemia are associated to an increase mortality, but in parallel ways.

The reduction of insulin resistance and consequent inflammation, could explicate our finding that the patients treated with metformin before hospitalization had a reduced risk of mortality during hospitalization.

Our data also demonstrated that an increase in basophil count was associated with a reduction in mortality. Only few works in the literature described the role of basophils in non-diabetic patients with COVID-19, despite their vital role in the pulmonary pathologies and regulation of immune responses, probably because their low number in the circulation is an important limitation. These studies suggested that basophils may exert a protective role; also, they showed that their absolute count seems to be reduced in COVID-19 patients as compared to controls, as well as in severe COVID-19 disease compared to mild/moderate disease [28]. Basophil cytokine responses to COVID-19 might help reducing the inflammation and also promoting antibody responses to the virus. Furthermore, basophils store the secretory granules of heparin that is only released into the vasculature at sites of injury [29], therefore helping maintaining a proper blood flow by balancing the active anticoagulant and procoagulant processes in pato-fisological condition. This property could be hypothesized also during the COVID-19 infection of diabetic patients. To our knowledge our study is the first that described a protective role of basophils in diabetic patients hospitalized with COVID-19, as they could act both reducing inflammation and improving an anticoagulant process.

An increased neutrophil count was found associated with an increased mortality in our cohort; we can theorize regarding the possible mechanism, since it is not clear and there are only few recent data on humans. Hyperglycemia affects the hematopoietic stem and progenitor cells in the bone marrow leading to enhanced myelopoiesis and elevated number of neutrophils and monocytes in the blood [30]. Moreover, neutrophils have been implicated in the induction of adipose tissue inflammation and insulin resistance; in fact, deletion of neutrophil elastase results in decreased adipose tissue inflammation, reduced myeloid cell content, and improved glucose tolerance and increased insulin sensitivity in obese mice [30]. Finally, we found an elevated neutrophil-to-lymphocyte (N/L) ratio, that can be due to an exaggerated myelopoiesis that typically elevates it [31].

Severe COVID-2019 disease is characterized by microthrombosis, increased coagulation and profound inflammation; platelets mediate thrombosis and, in these patients could increase thrombotic or inflammatory profile [32]. Consumption of platelets into a growing thrombus or platelet apoptosis might explain the thrombocytopenia present in patients with COVID-19.

An important and pragmatic aspect of our work is that the biomarkers employed can be obtained by the emergency laboratory in less than an hour and they are in-expensive and frequently used also in developing countries. Standardized protocols, well defined range limits, quality internal and external controls and low costs compared to research assay make them more interesting for characterize diabetic patients hospitalized for COVID-19 [33, 34].

Finally, we evaluated the pre-hospital status of our study population. Patients with microvascular complications and chronic kidney disease have higher mortality during hospitalization. Interestingly, no association of the other variables (macrovascular complications or obesity) before hospitalization with clinical outcomes has been evidenced, despite diabetes and cardiovascular disease are frequent comorbidities in patients with COVID-19 and play a role in adverse outcomes. Moreover, considering complications during hospitalization only respiratory failure increased mortality but singularly cardiovascular, thromboembolic or neurological complications did not.

The impact of sitagliptin treatment on mortality in diabetic patients and COVID-19 has been debated in literature [35, 36]; however we have only seven patients on sitagliptin in our cohort and therefore no analysis has been done.

One question of interest is the role of past glycemic control and not only acute hyperglycemia on the outcomes. In our study HbA1c before hospitalization were not associated with the clinical outcomes.

Our study has several limitations. Firstly during the pandemic COVID 19, hospital admissions increased sharply and the hospital rapidly became overloaded with patients affected by pneumonia and respiratory failure, of whom a relevant proportion in need of ICU admission and artificial ventilation. Intensive care unit (ICU) beds and ventilators were not available for all of these patients, therefore data relative to these outcomes could be underestimated.

Secondly, our data contain a high number of missing values of HBA1c, due to the fact that patients were not regularly followed by our outpatient service but by general practitioners.

Thirdly the current study was retrospective, with all the inherent limitations of such studies.

In conclusion hyperglycemia at admission, renal function and inflammatory parameters were found to be predictors of in-hospital mortality, while an increased basophil count was protective. Hyperglycemia had a direct effect on mortality, the indirect effect was only through absolute neutrophil count, CRP and PCT and markedly lower than the direct one.

## Data Availability

The data that support the findings of this study are available from the corresponding author, [ER], upon reasonable request.
